# Apical hypertrophic cardiomyopathy with preexcitation presenting as a myocardial infarction and ischemic stroke with a history of recurrent syncope: A case report

**DOI:** 10.1002/ccr3.2104

**Published:** 2019-03-14

**Authors:** Jonathan Al‐Saadi, Gustav Mattsson, Rozh Kader, Peter Magnusson

**Affiliations:** ^1^ Centre for Research and Development Uppsala University/Region Gävleborg Gävle Sweden; ^2^ Karolinska Institutet Stockholm Sweden; ^3^ Medical University of Gdansk Gdansk Poland; ^4^ Cardiology Research Unit, Department of Medicine Karolinska Institutet Stockholm Sweden

**Keywords:** apical, case report, hypertrophic cardiomyopathy, preexcitation, risk stratification, stroke

## Abstract

Contrast‐enhanced echocardiography or cardiac magnetic resonance imaging is of value in the diagnosis of apical hypertrophic cardiomyopathy. Apical hypertrophic cardiomyopathy is rare in Caucasians, and gene negativity does not rule out the diagnosis. Risk stratification for sudden cardiac death and decisions about anticoagulation in cases with atrial fibrillation should be based on guidelines.

## 1 INTRODUCTION

Hypertrophic cardiomyopathy (HCM) is a heterogeneous disease in several aspects, that is diagnosed when myocardial thickness is at least 15 mm, in the absence of abnormal loading conditions.^1^ Here, we highlight the difficulties of imaging and how apical HCM can be overlooked on transthoracic echocardiography without contrast. Furthermore, we give a practical example of risk stratification and anticoagulative treatment in patients with HCM and arrhythmias. We present a case with challenging diagnostic and therapeutic management. For a timeline of events see Table 1.

**Table 1:** Timeline

Time	Event
2015	Admitted due to chest pain.
	Transthoracic echocardiography: Hypertrophy.
	Bicycle exercise test ECG: Pathological reaction with ST depression.
2016	Syncope and frequent palpitations.
	Myocardial scintigraphy: Low reversibility of perfusion and low isotope uptake.
	24‐hour Holter monitoring: A supraventricular tachycardia of 7 s.
2017	Syncope and frequent palpitations.
	Implantation of insertable cardiac monitor
2018	Electroencephalogram: Normal.
Admission	Chest pain and palpitations.
Day 0	ECG: Generalized ST depression and discordant T‐waves.
	Coronary angiography: No significant stenosis.
	Signs of stroke
	Computerized tomography of cranium, aorta and coronary angiogram: Unremarkable.
Day 2	Cranial magnetic resonance imaging: Several infarctions in the mesencephalon and frontal cortex.
	Transthoracic echocardiography with contrast: Appearance consistent with apical HCM.
	Re‐examination of the ECG: Short PR interval and delta‐waves indicating preexcitation.
Day 6	Cardiac magnetic resonance: Confirmed the diagnosis of apical HCM.
	Insertable cardiac monitoring interrogation: Atrial fibrillation.

## 2 CASE HISTORY

A 63‐year‐old Caucasian woman was admitted to the emergency room due to chest pain and palpitations. Her medical history included at least two unexplained syncope episodes, frequent palpitations, and left ventricular (LV) hypertrophy believed to be secondary to hypertension. However, a recent echocardiography was interpreted as regression of the LV hypertrophy. Two years prior to admission, she had performed a bicycle exercise test ECG which showed a pathological reaction with a maximum ST depression of 3.1 mm. Furthermore, an earlier myocardial scintigraphy had shown low reversibility of perfusion and low isotope uptake in the basal and apical region of the heart. To evaluate the palpitations and syncope further, the patient had been equipped with an insertable cardiac monitor (ICM). Her mother died at 55 years of age from unexplained sudden cardiac death and the father at an age of 72 years from myocardial infarction.

**Figure 1:**
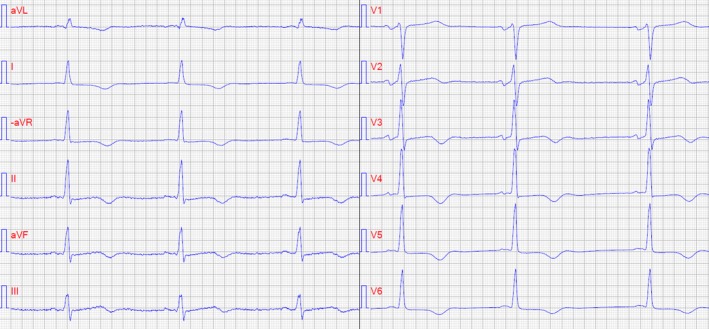
A 12‐lead ECG showing typical signs of preexcitation with short PR interval (<100 ms) and delta‐waves most prominent in aVF and III and typical signs of hypertrophic cardiomyopathy with generalized ST depression with inverted T‐waves in anterolateral leads. Paper speed 50 mm/s

The ECG showed sinus rhythm with ST depressions in leads as follows: II, aVF, and III and discordant T‐waves in all leads except for lead V_1_ and V_2_ in coherence with previous findings. A short PR time and delta‐waves were present, but not noted at the time (Figure 1). Troponin‐T series was elevated, 22, 134, and 525 ng/L, respectively. Coronary angiography was unremarkable except for atheromatosis of the left anterior descending artery. Myocardial infarction with nonobstructive coronary arteries (MINOCA) was considered. However, an hour after the coronary angiography the patient developed diplopia, anisocoria, and poor balance with a falling tendency to the left. Cranial computerized tomography (CT) showed no bleeding or signs of recent infarction. CT angiogram of the neck revealed no significant stenosis, occlusion or dissection of the vertebral and carotid arteries. CT of the aorta showed no dissection or rupture. However, cranial magnetic resonance imaging revealed several infarctions in the mesencephalon and frontal cortex, which were believed to be due to cardiac embolization.

**Figure 2:**
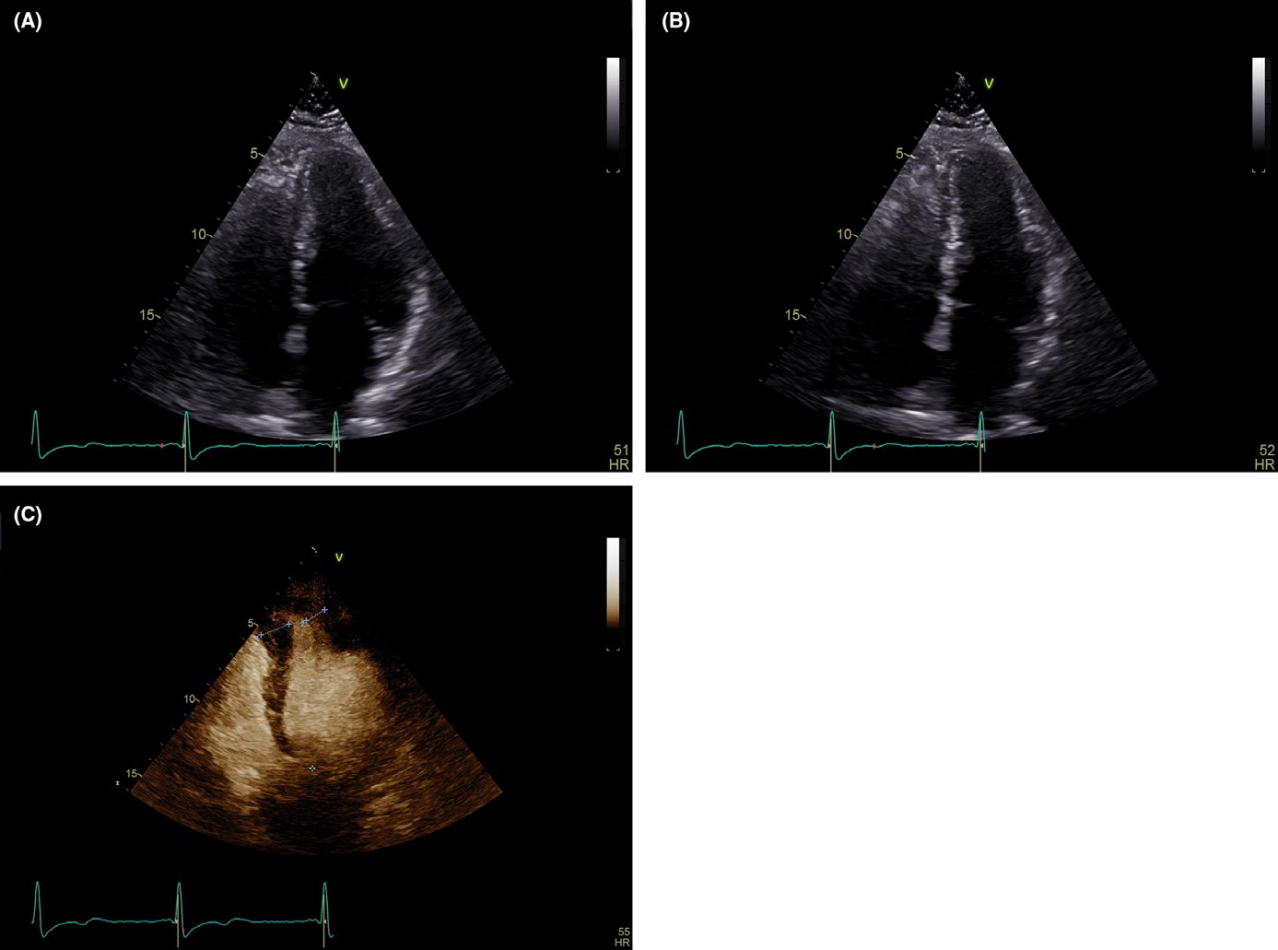
Transthoracic echocardiography in end‐diastole and end‐systole in the apical 4‐chamber view (A and B) Echocardiography with microbubble contrast revealing apical left ventricular hypertrophy (C)

**Figure 3:**
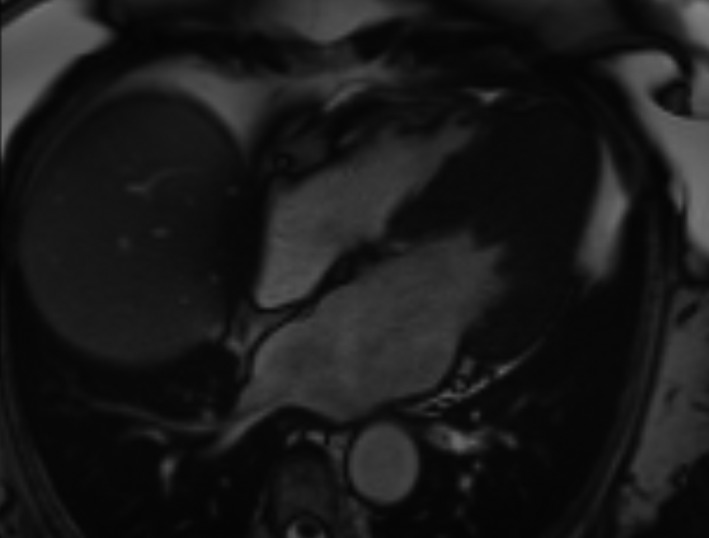
Magnetic resonance imaging of the heart displaying hypertrophic apical and inferolateral septum, appearance consistent with apical hypertrophic cardiomyopathy

A transthoracic echocardiogram (Figure 2) showed a LV end‐diastolic diameter of 5.1 cm and LV systolic function visually appeared normal, but impaired movement was seen in the apical region. The left atrial size diameter in parasternal axis measured 44 mm and maximal LV outflow gradient was 4 mm Hg. Importantly, with contrast, it revealed marked apical LV hypertrophy with a wall thickness of 17 mm, and appearance was consistent with apical HCM. The diagnosis was confirmed with a cardiac magnetic resonance (CMR) imaging which showed a LV mass of 175 g, end‐diastolic volume of 122 mL, and an ejection fraction of 74%. Moreover, it showed a hypertrophic apical and inferolateral septum, measuring at its widest point 17 mm with signs of edema and fibrosis, thus confirming the diagnosis (Figure 3).

The ICM interrogation revealed recurrent episodes of atrial fibrillation (AF). The patients CHA_2_DS_2_VASc score was 4 due to hypertension, female sex, and ischemic stroke. Re‐examination of the ECG and symptoms revealed preexcitation, which was attributed to the HCM diagnosis. She was discharged with dabigatran 110 mg twice daily and metoprolol succinate 100 mg once daily. The genetic analysis of the patient revealed no mutations; however, family members were given the opportunity of screening with echocardiography, and the patient was followed up at a rehabilitation center.

## 3 DISCUSSION

The prevalence of HCM is 0.2% and is similar between different racial groups but the apical form is more common in Japan (15%) compared to the United States (2%).^1^, ^2^

### 3.1 Diagnostics

The diagnosis of HCM requires a myocardial thickness of at least 15 mm.^1^ If abnormal loading conditions solely explain the increased wall thickness, which is then often concentric rather than asymmetric, the hypertrophy is deemed secondary. Apical HCM is characterized by myocardial hypertrophy predominantly in the left ventricular apex.^2^, ^3^ In this patient, neither hypertension nor valve lesions could explain the myocardial hypertrophy because repeated blood pressure measurements were considered normal and she had no confirmed aortic stenosis.

Furthermore, ECG may provide diagnostic clues; however, this was originally overlooked in this case; R/S amplitudes suggestive of hypertrophy, ST‐segment abnormalities, negative T‐waves, and pathological Q‐waves.^1^ In the absence of ischemic heart disease and/or heart failure, the ECG pattern with negative T‐waves was highly suggestive of underlying HCM. Prominent negative T‐waves in the precordial leads and/or inferolateral leads imply involvement of the apical region.^4^, ^5^

### 3.2 Genetic aspects on differential diagnostics

Up to 70% of HCM cases can be explained by genetic abnormalities, the vast majority sarcomeric protein genes (60%) but inborn errors of metabolism, neuromuscular disease, malformation syndromes, and amyloidosis explain 5%‐10%. Anderson‐Fabry disease accounts for 0.5%‐1.0% of patients diagnosed with HCM and often has clinical features such as angiokeratoma, hypohidrosis, paresthesia/sensory abnormalities, and neuropathic pain.^6^, ^7^ The next‐generation sequencing technique cover more than 99% of nucleotide substitutions associated with PRKAG2 and the X‐linked GLA and LAMP‐2 mutations.^1^ Thus, in the remaining patients, the phenotype may not be explained by a genetic abnormality.^1^

### 3.3 Imaging challenge

Echocardiography is limited by differences in interpretations between examiners and poor visualization of some segments of the heart. Apical hypertrophy is easily unnoticed due to near‐field artifacts and poor visualization of the lateral LV wall and apex. Interestingly, this patient was reevaluated using an ultrasound contrast agent, which revealed an apical form of HCM. The limitations of echocardiography and the important role CMR imaging have been demonstrated, especially in the presence of apical HCM.^3^, ^8^ CMR imaging and transthoracic echocardiography are currently recommended as initial diagnostic imaging and assessment. However, without the suspicion of apical HCM or careful judgement of inappropriate images, no further evaluation may be undertaken in clinical practice.^1^

### 3.4 Atrial fibrillation and risk of stroke

In a review of 33 studies, the prevalence of AF in HCM was 22% and the incidence of thromboembolism in HCM patients with AF was 3.8% per 100 patients per year.^9^ In fact, the European society of cardiology (ESC) states that *all* AF patients (paroxysmal, persistent, permanent) with HCM should be offered anticoagulation regardless of CHA_2_DS_2_‐VASc score.^1^, ^10^ Even in patients with implantable cardioverter defibrillators (ICDs), with capability to monitor AF, stroke is a leading cause of death.^11^

Several episodes of high atrial rate were detected by the ICM, which can be used for detection of AF.^12^ Despite limited evidence of the benefit of anticoagulation based on device‐detected AF,^13^ especially shorter episodes, given the guidelines that for HCM advocate munificent treatment, we justified dabigatran in this patient who already had suffered from a stroke.

### 3.5 Risk stratification of sudden cardiac death

Current risk stratification of sudden cardiac death takes age, left atrial diameter, LV outflow gradient, presence of nonsustained ventricular tachycardia, unexplained syncope, and family history of sudden cardiac death into account.^14^

Her mother did not have a confirmed HCM and was older than 40 years at the time of death. Thus, the calculated 5‐year risk was 2.66%, *ICD generally not indicated.*

If a nonsustained ventricular tachycardia would be detected, this would change to 5.97% which warrants an ICD. Thus, continuous monitoring of ventricular arrhythmias is advisable.

Preexcitation in HCM is rare; in one study, an accessory atrioventricular pathway was present in 5% of patients undergoing an electrophysiological study; the prevalence in unselected patients with apical HCM is unknown.^15^ In patients with preexcitation, treatment with prophylactic ablation is generally considered indicated if the RR interval is 240 ms However, in this case, the patient had a far longer refractory period during AF and thus low risk. In addition, she had no LV obstruction which makes her less prone to severe symptoms during rapidly conducted AF.

## 4 CONCLUSION

The combination of apical HCM without genotype and preexcitation is rare in Caucasians. This case emphasizes the importance of careful diagnostic work‐up including contrast echocardiography, anticoagulation, and genetic analysis. Moreover, it highlights the usefulness of an insertable cardiac monitor in detection of atrial arrhythmias and potentially nonsustained ventricular tachycardia for risk stratification.

## CONFLICT OF INTEREST

Dr Magnusson received an unrestricted research grant from Abbott and speakers fee from Alnylam, Bayer, Boehringer‐Ingelheim, Pfizer, and Novo Nordisk.

## AUTHOR CONTRIBUTIONS

JA: collected the data and involved in major writing and patient management. GM: wrote the manuscript and involved in project management. RK: collected the data, wrote the manuscript, and involved in patient management. PM: involved in idea, conceptualization, patient management, major writing, and project management. All authors read and approved the final manuscript.
